# Recapitulation of morphogenetic cell shape changes enables wound re-epithelialisation

**DOI:** 10.1242/dev.107045

**Published:** 2014-05

**Authors:** William Razzell, Will Wood, Paul Martin

**Affiliations:** 1 Schools of Biochemistry and, Physiology and Pharmacology, Faculty of Medical and Veterinary Sciences, University of Bristol, University Walk, Bristol BS8 1TD, UK; 2 Department of Biology and Biochemistry, University of Bath, Claverton Down, Bath BA2 7AY, UK

**Keywords:** Wound healing, *Drosophila* embryo, Actomyosin behaviour

## Abstract

Wound repair is a fundamental, conserved mechanism for maintaining tissue homeostasis and shares many parallels with embryonic morphogenesis. Small wounds in simple epithelia rapidly assemble a contractile actomyosin cable at their leading edge, as well as dynamic filopodia that finally knit the wound edges together. Most studies of wound re-epithelialisation have focused on the actin machineries that assemble in the leading edge of front row cells and that resemble the contractile mechanisms that drive morphogenetic episodes, including *Drosophila* dorsal closure, but, clearly, multiple cell rows back must also contribute for efficient repair of the wound. Here, we examine the role of cells back from the wound edge and show that they also stretch towards the wound and cells anterior-posterior to the wound edge rearrange their junctions with neighbours to drive cell intercalation events. This process in anterior-posterior cells is active and dependent on pulses of actomyosin that lead to ratcheted shrinkage of junctions; the actomyosin pulses are targeted to breaks in the cell polarity protein Par3 at cell vertices. Inhibiting actomyosin dynamics back from the leading edge prevents junction shrinkage and inhibits the wound edge from advancing. These events recapitulate cell rearrangements that occur during germband extension, in which intercalation events drive the elongation of tissues.

## INTRODUCTION

Throughout embryonic development, individual epithelial cells divide, stretch and rearrange in a concerted way to force epithelial sheets to bend and sweep forwards during the morphogenetic episodes that sculpt embryonic shape (Guillot and Lecuit, 2013). It is likely that aspects of this tissue-building machinery may be reactivated following tissue damage to repair an epithelial wound (Martin and Parkhurst, 2004). Indeed, after wounding simple epithelia, as in embryonic tissue or adult cornea or gut, the leading edge cells assemble an actomyosin ‘purse-string’, which draws the epithelial hole closed (Brock et al., 1996; Danjo and Gipson, 1998; Martin and Lewis, 1992; Wood et al., 2002). This contractile cable and the associated dynamic filopodia recapitulate the actin machineries that lead to dorsal closure in the *Drosophila* embryo (Jacinto et al., 2002b). However, cells back from the leading edge contribute also. Indeed, in adult mammalian tissues, cells up to 30-40 rows back from the advancing wound edge become involved in the repair process (Matsubayashi et al., 2011; Meyer et al., 2012; Werner et al., 1994). In this study, we analyse the shape changes that occur in front row cells and those several cell rows back in wounds made in the *Drosophila* embryo epidermis. We show that multiple rows of cells stretch towards the wound, and that the junctions between rows of cells lying anterior-posterior to the wound margin shrink, leading to subsequent intercalation episodes that resemble those of germband extension. These junction shrinkage and intercalation events are associated with, and dependent upon, myosin-II pulses and enable the cable to efficiently drag the wound edge forward.

## RESULTS AND DISCUSSION

**Fig. 1.**
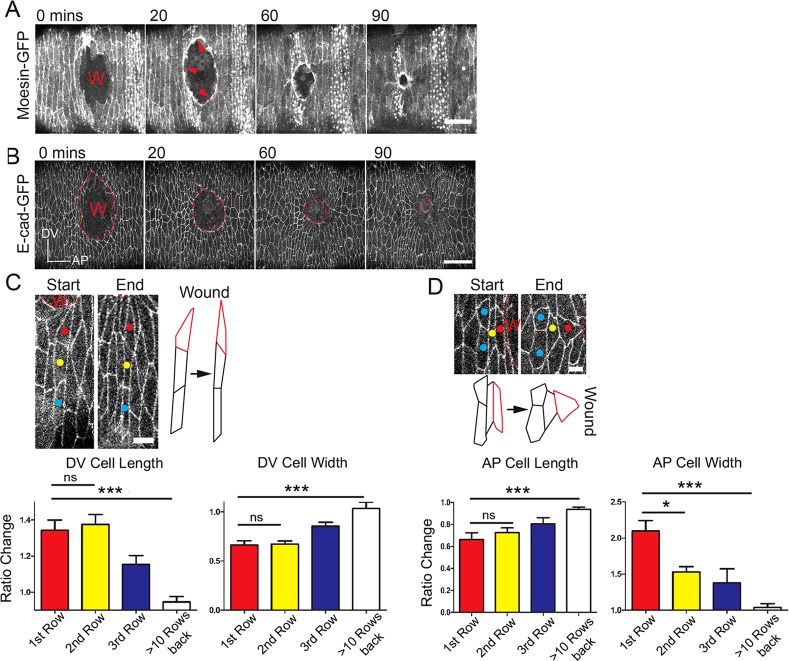
**Multiple cell rows stretch towards the closing wound.** (A) A time course series of wound closure in a Moesin-GFP-expressing embryo illustrating the actomyosin ‘purse-string’ (red arrows). (B) An equivalent E-cadherin-GFP wounded embryo showing cell shape changes at the wound edge. Axis inset shows dorsal-ventral (DV) and anterior-posterior (AP) orientation. (C) High magnification views of start and end images from a wounded E-cadherin-GFP embryo and accompanying schematics to highlight cell shape changes of DV cells (C) versus AP cells (D). Cells at the immediate wound margin outlined in red. Graphs represent relative length and width of cells at the end versus start of wounding (*n*≥12 cells for each row from three wounds). Wounds (W) marked by the dashed lines. Error bars represent s.e.m. Scale bars: 20 µm (A); 25 µm (B); 5 µm (C,D). Time is in minutes. ns, not significant, *P<0.05, ***P<0.001, one-way ANOVA with Bonferroni's post-hoc test.

As previously shown, wounding the ventral epidermis of stage 14 *Drosophila* embryos with a laser leads to relaxation of adjacent tissue such that the wound gapes open leaving an ovoid defect that reflects inherent tissue tensions (Fig. 1A) (Hutson et al., 2003; Wood et al., 2002). Subsequently, the wound hole is drawn closed by an actomyosin cable that rapidly assembles in the wound edge cells and contracts a small wound with, for example, initial diameter of 30 µm to within 5% of its original area within 90 min of wounding (Fig. 1A). Expression of *Drosophila* E-cadherin-GFP (Oda and Tsukita, 2001) enables us to observe the cell shape changes that occur during healing.

### Cells back from the wound edge change their shape in ways that reflect local tissue tensions

Besides assembling a contractile actomyosin cable and actin-rich protrusions, the front row cells extend towards the wound, as do cells several rows behind them (Fig. 1B). Front row cells that lie dorsal-ventral to the wound (DV cells) become longer by 1-2 µm or 30% of their original length (Fig. 1C), but this alone is not sufficient to close the wound. As with dorsal closure, in which multiple cell rows back from the leading edge elongate to enable epithelial hole closure (Jacinto et al., 2002a; RiesgoEscovar and Hafen, 1997), we see several rows of wound edge cells stretching. However, cells lying anterior-posterior to the wound must extend in their short axis to contribute to repair. These cells (AP cells) extend in width by twofold as they are drawn forwards by the contracting actin cable (Fig. 1D).

### Cells anterior-posterior to the wound edge show specific junction shrinking in a pulsatile manner that leads to cell intercalations

**Fig. 2.**
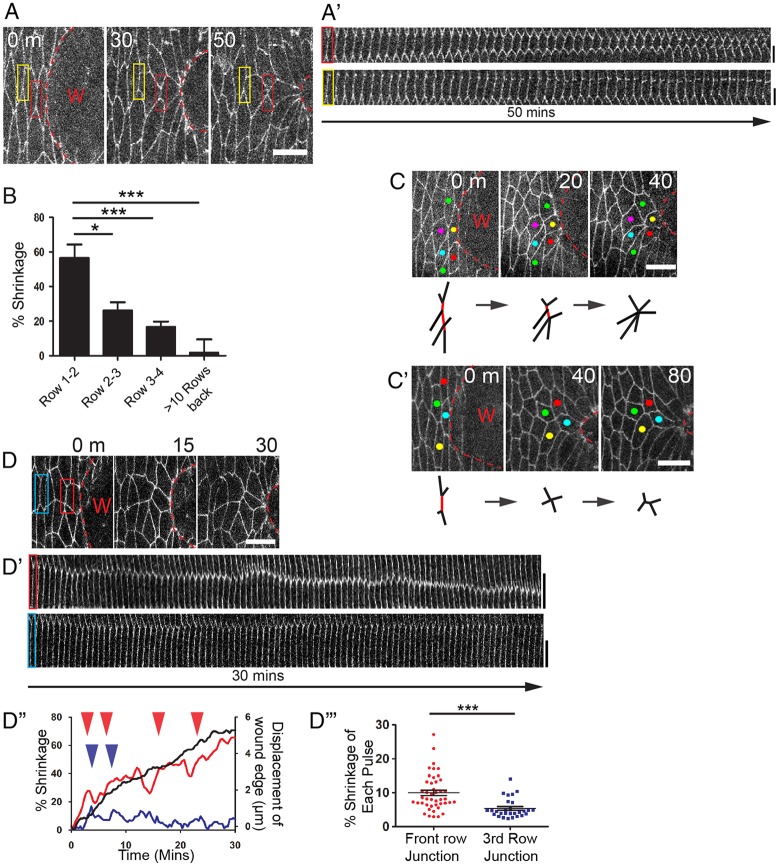
**AP cells exhibit ratchet-like junction shrinking and cell intercalation events.** (A) Time-lapse (still) images from a wounded E-cadherin-GFP embryo showing a junction between two AP cells (red box) shrinking as the wound closes. (A′) Kymograph analysis of the junctions highlighted by the red and yellow boxes in A shows junctions shrinking over time. (B) Graph illustrating percentage junction shrinkage over 60 min versus junctional distance back from the wound edge (*n*≥17 junctions from six wounds for each row). (C,C′) Example of the shrinking of multiple adjacent junctions to form a multicellular rosette (C) and shrinking of a junction leading to an intercalation event (C′). Schematics beneath indicate shrinking junctions in red. (D,D′) A time-lapse series of (still) images of AP cells (D) used for kymograph analysis (D′) of the junctions highlighted by the red and blue boxes in D. (D′′) Plot of wound edge advancement (black) and percentage junction shrinking between wound edge cells (red) and far back from the wound edge (blue) as highlighted in D. (Arrowheads indicate pulses of shrinking.) (D′′′) Plot showing contraction pulses in cells positioned various distances from the wound edge (*n*≥14 junctions from six wounds for each). Wounds (W) highlighted by the dashed lines in all. Error bars represent s.e.m. Scale bars: 10 µm (A,C,C′,D); 5 µm (A′,D′). Time is in minutes. *P<0.05,***P<0.001, one-way ANOVA with Bonferroni's post-hoc test (B) or Student's *t*-test (D′′′).

We wondered whether other mechanisms are used by AP cells to enable release of tension and stretching of the epithelium in response to contraction of the actin cable. Observation of these cells over the wound repair period reveals a dramatic shrinking of junctions that are perpendicular to the pulling force of the wound (Fig. 2A). Kymograph analysis (Fig. 2A′) shows that shrinkage of the junctions linking rows one and two was 57%, and that further back, between the second and third, and between the third and fourth rows, the junctions shrank by 26% and 16%, respectively (Fig. 2B). During the same period, cells distant from the wound edge show no change in length (Fig. 2B), although it has been shown that over a longer time course of several hours, junction shrinking does contribute to alignment of denticle field cells into the parallel arrays seen in stage 14 epidermis (Simone and DiNardo, 2010).

At the wound edge, shrinking is often not confined to an individual junction or pair of cells. We observe several neighbouring junctions in the same row shrinking concurrently to a single point to give rise to a multicellular rosette (Fig. 2C). Single junctions that shrink completely give rise to new junctional associations between previously non-neighbouring cells (intercalating tetrads) and these new junctions always lie parallel to the closing wound (Fig. 2C′). During the longer 3 h closure of large wounds (∼80 µm across), one in 20 junctions were observed to shrink as part of linked junctions resulting in rosette formation, and one in ten shrunk resulting in intercalating tetrads. In unwounded embryos, only one in 307 and one in 44 junctions were involved in similar rearrangements, respectively (*n*=3 wounds; supplementary material Fig. S1Ai-Bii). Cell rosettes and intercalating tetrads were observed up to eight rows back from the edge of large wounds.

The cell shape changes that drive these cell intercalations within the advancing epithelial wound sheet recapitulate similar events that occur during the developmental process of germband extension (supplementary material Fig. S1Ci,ii), whereby junction shrinking and resolving in the direction of the elongating tissue is believed to drive tissue extension (Bertet et al., 2004; Blankenship et al., 2006; Butler et al., 2009; Fernandez-Gonzalez et al., 2009). During wound closure, the same events may actively contribute to the wound epithelial advance with a ‘pushing’ force or, rather, may be a passive, enabling response to the actomyosin cable contractile forces from the front row cells.

### Pulses of junction shrinking correlate with pulses of myosin directed to the cell junctions

To investigate the dynamics of junction shrinkage, we made movies of wounded E-cadherin-GFP-expressing embryos at high temporal resolution (20 s intervals; Fig. 2D; supplementary material Movie 1). Kymograph analysis reveals that shrinkage occurs in a pulsatile manner (Fig. 2D′), with these contractions being significantly larger than the small transient fluctuations seen in cells away from the wound (Fig. 2D′-D′′′). Furthermore, the number of contractions correlated with the extent of overall junction shrinkage (supplementary material Fig. S1Di,ii), indicating that the pulses may drive junction shrinking in wound edge cells. Junction shrinking does not appear to be a direct, passive consequence of actomyosin cable contraction because wound edge advancement was relatively smooth compared with the ratchet-like fluctuations in junction size (Fig. 2D′′).

**Fig. 3.**
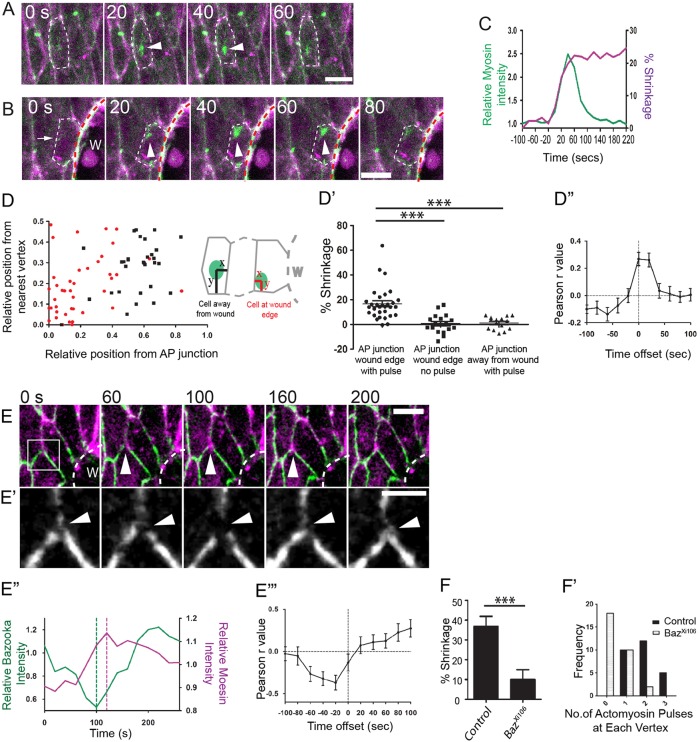
**Junctional shrinking pulses correlate with myosin flashes near the junction.** (A,B) Time-lapse still images showing myosin (green, arrowheads) coalescing at the apex of a cell in the unwounded epithelium (A) and at the vertex of a wound edge cell (arrowheads) of an AP junction (arrow) (B). Dashed lines indicate cell outlines. (C) Plot of relative myosin intensity (green) versus percentage shrinking of the AP junction (magenta) over time. (D) Plot of the location of myosin pulses (indicated by green in schematics) in cells at wound edge (red) versus at least three rows away from the wound (black) (*n*≥25 pulses from 21 cells from eight different wounds for each cell type). (D′) Plot of percentage AP junction shrinkage after each pulse of myosin (*n*≥18 pulses from 15 cells in ten wounds for each). (D′′) Pearson correlation for the change in myosin intensity around the shrinking AP junctions versus change in junction length. *r* values were calculated by shifting the myosin data set in time (*n*=22 junctions from ten wounds). (E) Time-lapse (still) images from a wound edge cell as Bazooka-GFP (green) is lost from the cell vertex (arrowheads) of the AP junction, immediately prior to an actin pulse (magenta). (E′) High magnification view of the region highlighted in E of the Bazooka-GFP channel only, showing a break and re-sealing of Bazooka (arrowheads). (E′′) Plot of the relative Bazooka-GFP intensity (green) and relative Moesin intensity at the break point in E. Dashed lines indicate where loss of Bazooka precedes an actin pulse. (E′′′) Pearson correlation of Bazooka-GFP intensity versus mCherry-Moesin intensity at cell vertices. *r* values were generated by shifting the Moesin data set in time (*n*=24 vertices from six wounds). (F) Percentage junction shrinkage over 30 min of wound closure in control versus *Baz^Xi106^* mutant embryos (*n*≥22 junctions from five wounds for each). (F′) Frequency distribution of actin pulses in control versus *Baz^Xi106^* mutant embryos (*n*≥27 vertices from six wounds for each). Wounds (W) all marked by dashed white lines. Scale bars: 5 µm (A,B,E); 2 µm (E′). Error bars represent s.e.m. ***P<0.001, one-way ANOVA with Bonferroni's post-hoc test (D′) or Student's *t*-test (F). Time is in seconds.

During germband extension, pulses of myosin-II at the junction precede shrinking of polarised membranes, which, in turn, drive cell intercalation events (Rauzi et al., 2010). To investigate whether a similar mechanism might be operating in the wound epithelium, we imaged myosin-II activity in unwounded tissue and compared it with that of wound edge cells in Spaghetti squash:GFP (regulatory light chain of myosin) fusion embryos. In unwounded tissue, concurrent actin-myosin ‘flashes’ can be seen coalescing at the apical surface of cells in a series of pulses, with a frequency of 0.62/min (±0.15 s.d., *n*=19 cells from eight embryos; Fig 3A; supplementary material Fig. S2Ai,ii). Regular actomyosin pulses in the unwounded epithelium may make cells responsive to occasional cues directing homeostatic shaping of the epithelium as required, for example, to maintain segment boundaries (Bulgakova et al., 2013; Marcinkevicius and Zallen, 2013). Actomyosin pulses in AP cells at the wound edge (and in second row cells), appeared to be associated with cell vertices of shrinking junctions as the cells are tugged by the closing wound (Fig. 3B,D; supplementary material Fig. S2B and Movie 3). The majority of myosin pulses occur next to a vertex of a shrinking junction, whereas in unwounded epithelium, pulses target the centroid of the cell preceding minor transient contractions of the apical cell area (Fig. 3A; supplementary material Movie 2); these episodes may be responsible for some of the small pulses of junction shrinking observed in cells back from the wound edge (Fig. 2D′′). Each pulse of myosin in wound edge cells is associated with significant junctional shrinkage (on average 16%, compared with 1.5% when myosin pulses occur in cells several rows back from the wound edge; Fig. 3D′). Furthermore, we measured a positive correlation between the change in myosin intensity and change in junction length (Fig. 3D′′). Shifting the data sets in time relative to each other revealed that junction shrinkage lags behind the myosin pulse by up to 20 s (Fig. 3D′′), which is similar to the lag reported for apical area constriction due to myosin activity in ventral furrow cells during *Drosophila* gastrulation (Martin et al., 2009).

During germband extension, myosin accumulates at junctions and is believed to stabilise junctions as they shrink (Rauzi et al., 2010). We saw no such accumulation in wound edge cells (supplementary material Fig. S2Di), but this may reflect the fact that wound cell rearrangements occur over a much longer time period than that during germband extension for which rapid tissue elongation may require stabilisation of junction shrinking. We observe occasional junctional rises in myosin intensity as myosin pulses developed in wound edge cells, but these rises generally resolved (supplementary material Fig. S2Dii). Recently, actomyosin pulses have been shown to drive transient wave-like apical contraction in cells after wounding the *Drosophila* pupal epidermis (Antunes et al., 2013), and they may also help drive rapid wound repair in stage 7 *Drosophila* embryos (Fernandez-Gonzalez and Zallen, 2013).

The localisation of myosin flashes near to cell vertices suggests that this region of the cell is responsible for sensing mechanical forces within the advancing epithelium. Indeed, it is known that myosin is recruited to sites of tension, for example during gastrulation (Pouille et al., 2009), germband extension (Fernandez-Gonzalez et al., 2009) and dorsal closure (Franke et al., 2005). To understand how myosin could be recruited to AP wound cell vertices/junctions, we imaged Bazooka-GFP which is excluded from myosin-rich anterior-posterior cell junctions during germband extension through its phosphorylation by Rho-associated Kinase (ROCK; Rok) (Simoes et al., 2010). Bazooka-GFP is localised cortically at cell-cell margins, but in cells that are tugged by the closing wound, we observe small, transient breaks at vertices of AP cell junctions (Fig. 3E,E′; supplementary material Movie 4). These breaks in Bazooka rapidly recruit a pulse of actin that precedes closure of the Bazooka break (Fig. 3E). At the wound edge, 16 of 24 vertices (from six wounds) exhibited at least one break and five of 24 showed more than one break in the 15 min of wound closure monitored (supplementary material Fig. S3Ai), whereas back from the wound edge no significant breaks were observed (supplementary material Fig. S3Aii). Only those junctions with a vertex with a Bazooka break underwent significant AP junction shrinkage (supplementary material Fig. S3Aiii). During the periods when breaks appeared, Bazooka-GFP intensity was negatively correlated with mCherry-Moesin intensity, and shifting the data sets relative to each other showed that the peak of Moesin intensity occurred 20 s after the Bazooka break (Fig. 3E′′′). We saw no E-cadherin-GFP breaks at wound edge vertices (*n*=9 vertices from three wounds; supplementary material Fig. S3B). The Bazooka breaks may be a read-out of where the tension from the pull of the actin cable is ‘sensed’ in the cell. We believe the breaks have some functional significance in recruiting actomyosin because *Bazooka^Xi106^* mutant hemizygous embryos exhibit much reduced junction shrinkage compared with heterozygous controls (Fig. 3F), as well as reduced actomyosin activity at the vertices (Fig. 3F′; supplementary material Fig. S3Ci,ii). Because of the parallels with germband extension, it is tempting to speculate that Rho1 signalling might link the mechanical forces from the pulling actin cable, as reported for AP cell edges during germband extension (Simoes et al., 2010).

### Blocking myosin dynamics and junction shrinking reduces wound edge advancement

**Fig. 4.**
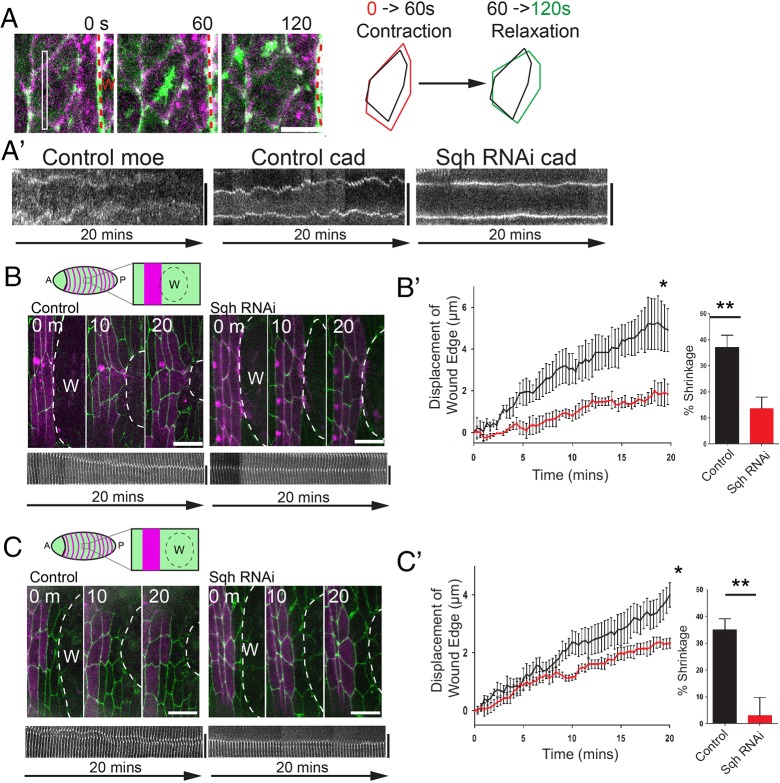
**Inhibiting myosin pulses prevents junction shrinkage and wound edge advancement.** (A) Time-lapse (still) images from a movie showing a myosin (green) pulse as it drives contraction and then relaxation (see schematic) of a wound edge cell. (A′) Kymograph analysis of cell areas showing apical area fluctuations in control cells from A (Control moe, Control cad), but no fluctuations when these cells express Spaghetti-squash RNAi (Sqh RNAi cad). (B,C) Wounds made immediately adjacent to control or Sqh RNAi-expressing engrailed cells (B) or with one row of wild-type cells between the wound and engrailed stripes (either control or expressing Sqh RNAi; C) (see schematics) in E-cadherin-GFP embryos with engrailed stripes labelled with mCherry-Moesin (magenta). Kymograph analysis shows junction dynamics in control and Sqh RNAi-expressing cells for junctions between the first and second row cells. (B′,C′) Plots of epithelial advancement over time in wounds made immediately adjacent to (B′) or with one row of wild-type cells in front of (C′) control and Sqh RNAi-expressing cells (*n*=3 wounds for each in B′ and *n*=7 in C′). Bar graphs show percentage shrinking of junctions between front and second row cells after 20 min of wound closure (*n*≥7 junctions from three wounds in B′ and *n* ≥7 junctions from seven wounds in C′). Error bars represent s.e.m. Time is in seconds (A) or minutes (B,C). Scale bars: 5 µm (A); 10 µm (B,C). *P<0.05, two-way ANOVA with Bonferroni's post-hoc test; **P<0.01, Student's *t*-test.

To test whether myosin pulses and the resulting junction shrinkage events could release epithelial tension and thus enable the actin cable to efficiently draw the wound epidermis closed, we expressed Spaghetti-squash RNAi (Sqh RNAi) in engrailed stripes (via *engrailed*-Gal4) to knockdown myosin activity (supplementary material Fig. S4A) and made small laser wounds (30 µm in diameter) immediately adjacent to these knockdown cells; these wounds did not encroach on more anterior engrailed stripes (supplementary material Fig. S4B). The stochastic small pulsations we see in cells back from the wound edge cease after expression of Sqh RNAi in engrailed stripes, confirming successful blockade of myosin (Fig. 4A,A′). Wounding immediately adjacent to the engrailed cells, such that knockdown cells form part of the leading edge, led to a delay in wound contraction and reduced shrinkage of the junctions between the first and second row of cells (Fig. 4B,B′). However, as myosin also has a role in local actin cable contraction, these junctions may be failing to shrink simply because of a diminished pulling force from the wound edge. We therefore wounded one cell row in front of the *engrailed*-expressing cells (Fig. 4C) so that leading edge cells are wild type and pull normally on Sqh RNAi neighbouring cells behind them. Leading edge actomyosin cable assembly was now normal (supplementary material Fig. S4C). In such cases, we again observe a reduction in wound edge advancement (Fig. 4C,C′) and reduced junction shrinking between the first and second row of cells (Fig. 4C′). Myosin is knocked down in the second row cells, but not in the front row, indicating that cells on both sides contribute to junction contraction (supplementary material Fig. S2B). In *Baz^Xi106^* mutants, in which junction shrinking was also reduced, wound edge movement was similarly inhibited (supplementary material Fig. S3D).

In this study, we show that cells back from the wound edge play a key role in repair by their oriented cell stretching and myosin-driven junction contractions leading to cell intercalation events that enable release of tension and wound re-epithelialisation.

## MATERIALS AND METHODS

### Fly lines

*Drosophila* were maintained on cornmeal molasses food at 23°C. Da-Gal4, UAS-GFP-Moesin or E22c-Gal4, UAS-mCherry-Moesin were used to label the actin cytoskeleton (Edwards et al., 1997; Wodarz et al., 1995; Dierick and Bejsovec, 1998). Ubi-E-cadherin-GFP (Oda and Tsukita, 2001) was used to follow cell shape changes. To analyse myosin dynamics in cells, Sqh-GFP was expressed from the *sqh* promoter on a *Sqh^AX3^* background (Royou et al., 2004, 1999). UAS Bazooka-GFP (Sanchez-Soriano et al., 2005) was expressed by E22c-Gal4. Spaghetti squash RNAi (TRIP^HMS00830^) was expressed in epithelial stripes by *engrailed*-Gal4. Lines were obtained from the Bloomington *Drosophila* Stock Center. Genotypes used are shown in supplementary material Table S1.

### Collecting and imaging embryos

Embryos were laid overnight at 23°C on apple juice agar dishes, dechorinated in bleach and mounted on Greiner Lumox gas-permeable culture dishes (Sigma) in halocarbon oil 700 (Sigma) or on glass slides with double-sided tape with Voltalef oil (VWR International). For experiments using RNAi, embryos were laid at 28°C. Embryos were wounded at stage 14 using a nitrogen ablation laser (Spectra-Physics) attached to a Zeiss Axioplan 2 widefield imaging system. Confocal imaging was performed on a Leica SP5-II confocal laser scanning microscope. Image preparation and analysis was performed on maximum projected confocal movies and utilised Volocity (PerkinElmer) and ImageJ (NIH) software. Graph plotting and statistical analysis were performed using Prism (Graphpad). Image quantification methods can be found in the supplementary material.

## Supplementary Material

Supplementary Material
