# Supplementary material for: Recapitulation of morphogenetic cell shape changes enables wound re-epithelialisation
Source: Development. 2014 May;141(9):1814–20. doi: 10.1242/dev.107045 (PMC3994776; doi:10.1242/dev.107045)
Supplement: Supplementary Material [file supp_141_9_1814__index.html]

Recapitulation of morphogenetic cell shape changes enables wound re-epithelialisation — Supplementary Material 

# Recapitulation of morphogenetic cell shape changes enables wound re-epithelialisation

## DEV107045 Supplementary Material

**Files in this Data Supplement:**

- **Supplementary Material**
