# Supplementary material for: Recapitulation of morphogenetic cell shape changes enables wound re-epithelialisation
Source: Development. 2014 May;141(9):1814–20. doi: 10.1242/dev.107045 (PMC3994776; doi:10.1242/dev.107045)
Supplement: Supplementary Material [file supp_141.9.1814_DEV107045.pdf]

Supplementary materials and methods

Image quantifications

Maximum projected confocal movies were used for quantification except when measuring Bazooka pulses when the stacks were summed. For measurements of cell shape changes, cell length refers to the dimension of the cell along the embryo's DV axis, and width refers to the dimension along the AP axis.

To quantify the pulses of junction shrinking, junction length was tracked manually from movies (commencing 10 minutes post-wounding) of wounds made to E-cadherin-GFP-expressing embryos. Pulses were considered to have occurred when the change in junction length exceeded one standard deviation above the mean change for three consecutive time points.

To plot the location of myosin pulses in wound edge cells relative to the AP junction, we measured the distance between the centre of each myosin pulse from the nearest vertex as a proportion of the length of the AP junction (*x*-axis). The same was performed along the *y*-axis to measure the pulse distance from the AP junction as a proportion of the cell width. For wound edge cells, the myosin pulses were measured relative to the AP junction between cell rows one and two. For cells back from the wound edge, measurements were made relative to the AP junction in these cells closest to the wound.

The junction shrinkage as a result of each myosin pulse was calculated by measuring the length of the junction one time point before the myosin pulse and again after the pulse had resolved. The Pearson r value of myosin pulses versus junction shrinkage was assessed by drawing a 3 μm ROI around the AP junction and using this to measure the Sqh-GFP average intensity. The junction shrinkage was tracked manually and the change in Sqh-GFP intensity and junction length between timepoints used to calculate the r value. The time shifts were achieved by shifting change in the myosin intensity data set relative to change in the junction length data set.

To measure Bazooka breaks and E-cadherin intensity at vertices, 15-minute periods of wound closure were analysed (movies started 20 minutes post-wounding). A 0.58 μm ROI was drawn around the vertex and used to measure average Bazooka-GFP intensity, and a 1.54 μm ROI to measure average mCherry-moesin intensity. The average intensities are expressed relative to a linear regression analysis of the data. A break was identified if the Bazooka-GFP intensity dropped one standard deviation of the data set below a relative Bazooka-GFP intensity of 1 for three consecutive time points. The intensity of Bazooka-GFP and mCherry-moesin during the break period and four time points either side was used to calculate Pearson r values. For identifying pulses of actin in Bazooka mutant embryos, GFP-moesin intensity was analysed in 20-minute movies, starting 20 minutes post-wounding. The GFP-moesin average intensity at cell vertices was monitored with a 2 μm ROI drawn around the vertex and expressed relative to the linear regression analysis of the data. GFP-moesin pulses were identified if the relative intensity exceeded one standard deviation above a relative GFP-moesin intensity of 1 for three consecutive time points.

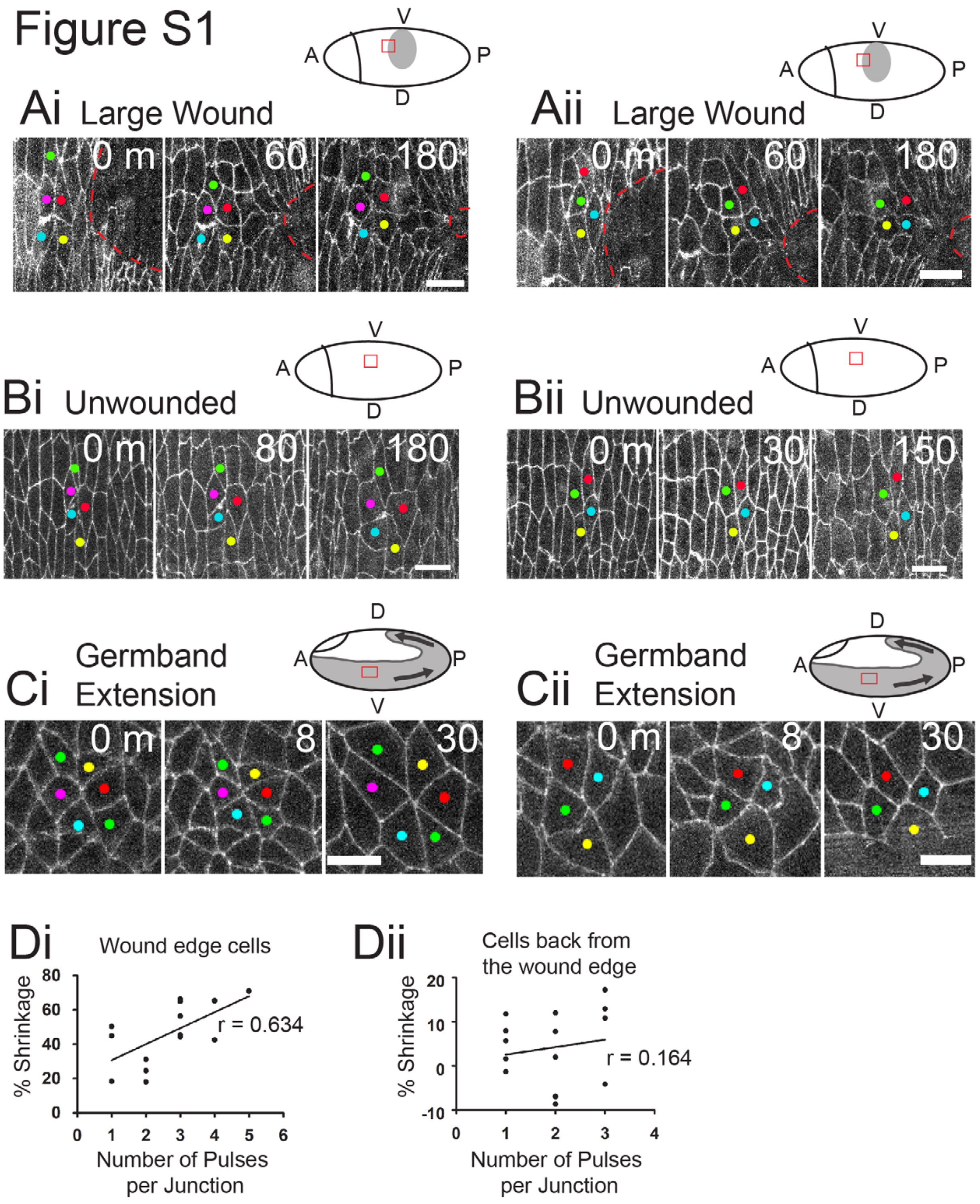

Supplementary Figure S1

(Ai) Timelapse stills of the wound edge of large, 80 μm, wounds made to E-cadherin-GFP expressing stage 14 embryos showing an example of multicellular rosette formation and analagous examples in (Bi) unwounded embryos and (Ci) in cells during germband extension at embryonic stage 8. (Aii) An equivalent wound edge to show an example of a single junction shrinking event leading to intercalation of a tetrad of cells and analagous examples in (Bii) unwounded embryonic epidermis and (Cii) in cells participating in germband extension. Schematics above each example show image area in red and wounds/ germband in grey. (Di) A scatter plot showing a strong positive correlation between the number of contractions identified at each junction at the wound edge and the overall shrinkage of the junction. The correlation is much less in cells back from the wound edge as shown in the scatter plot in (Dii) ($n \geq 14$ junctions from 6 wounds for each). r represents a Pearson correlation generated from the linear regression analysis. Scale bars represent 10 μm in all. Times are in minutes for all.

Figure S2

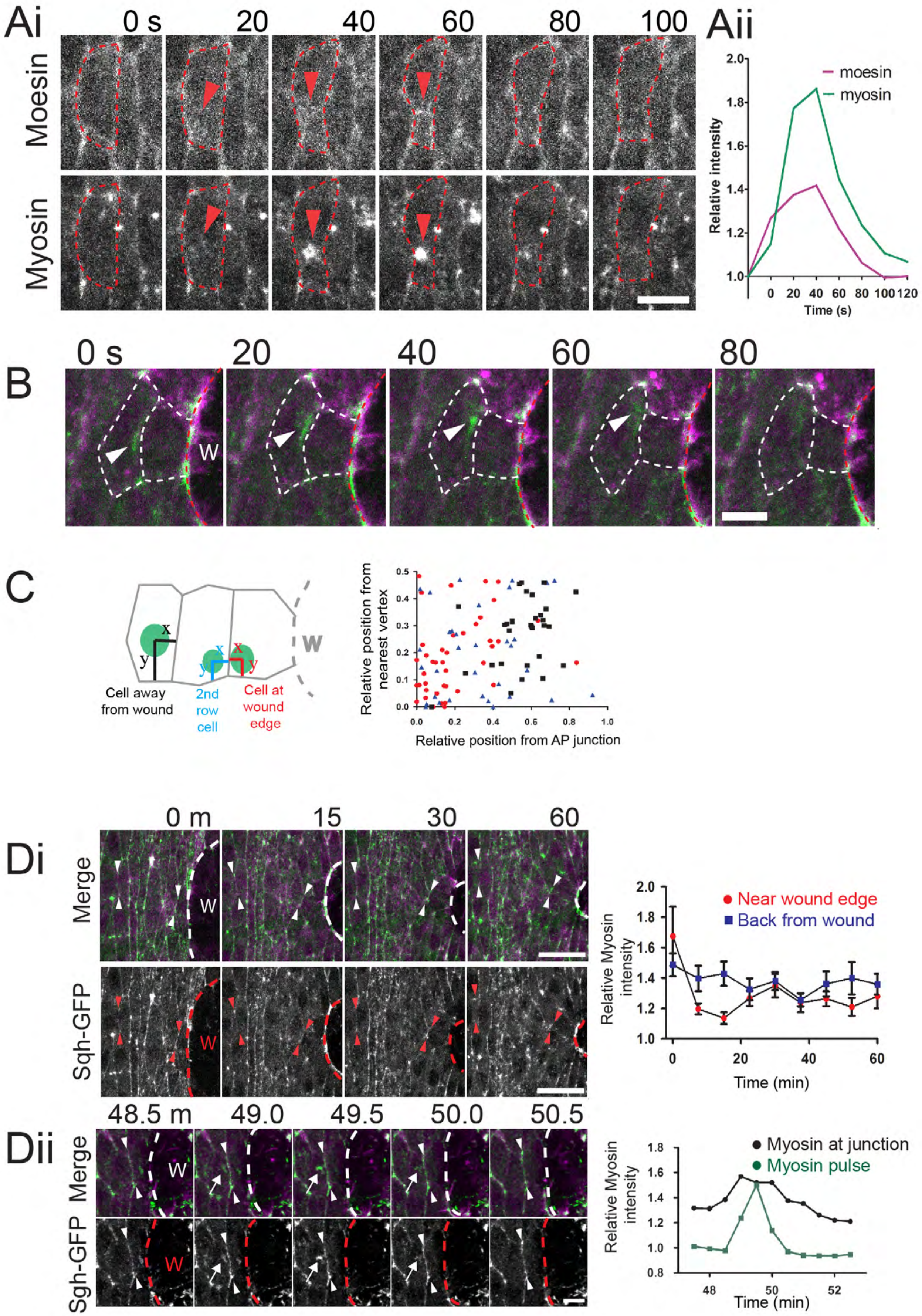

Ai
0 s
20
40
60
80
100
Moesin
Myosin
Aii
moesin
myosin
Relative intensity
Time (s)
B
0 s
20
40
60
80
W
C
Cell away from wound
2nd row cell
Cell at wound edge
W
Relative position from nearest vertex
Relative position from AP junction
0 m
15
30
60
Di
Merge
Sgh-GFP
W
Near wound edge
Back from wound
Relative Myosin intensity
Time (min)
48.5 m
49.0
49.5
50.0
50.5
Dii
Myosin at junction
Myosin pulse

Supplementary Figure S2

(Ai) A cell in unwounded tissue showing that moesin (actin) dynamics mirror myosin dynamics suggesting that actin and myosin are recruited together. (Aii) Plot of relative intensity of moesin and myosin in the cell in Ai. (B) Stills from a confocal movie showing myosin pulses (green) in a second row cell recruited to the AP junction between cells in rows one and two. Arrow points to the myosin pulse; the wound (W) is marked by the red dashed lines and the cell outlines by white dashed lines. (C) Plot of the location of myosin pulses (indicated by green in schematics) from the graph in Figure 3Di with addition of pulses from cells in the second row (blue) (n = 33 myosin pulses from 17 cells from 9 different wounds). (Di) Stills from a movie of the wound edge in an mCherry-moesin (magenta), Sqh-GFP (green, grey in bottom panel) expressing embryo showing no significant accumulation of myosin at the junctions of cells at the wound edge, nor further back from the wound (examples of the junctions followed are highlighted by arrowheads). Graph represents average Sqh-GFP intensity at junctions relative to that in the cell over 60 minutes of wound closure ($n \geq 12$ junctions from 5 movies for each). (Dii) Close up still images from a time-lapse of a junction at the wound edge of an mCherry-moesin (magenta), Sqh-GFP (green, also shown in the bottom panel, grey) expressing embryo showing that individual junctions may accumulate myosin during myosin pulses, but this subsequently resolves (the junction observed is highlighted by the arrowheads, and the myosin pulse by the arrow). Graph shows the intensity of myosin at the junction and in the myosin pulse relative to the whole cell intensity, highlighted by the arrowheads/ arrows in the adjacent still images. Wound (W) margins marked by dashed lines. Error bars represent SEM. Scale bars represent 5 µm in all except 15 µm in Di. Time is in seconds for Ai-B and minutes for Di-ii.

Figure S3

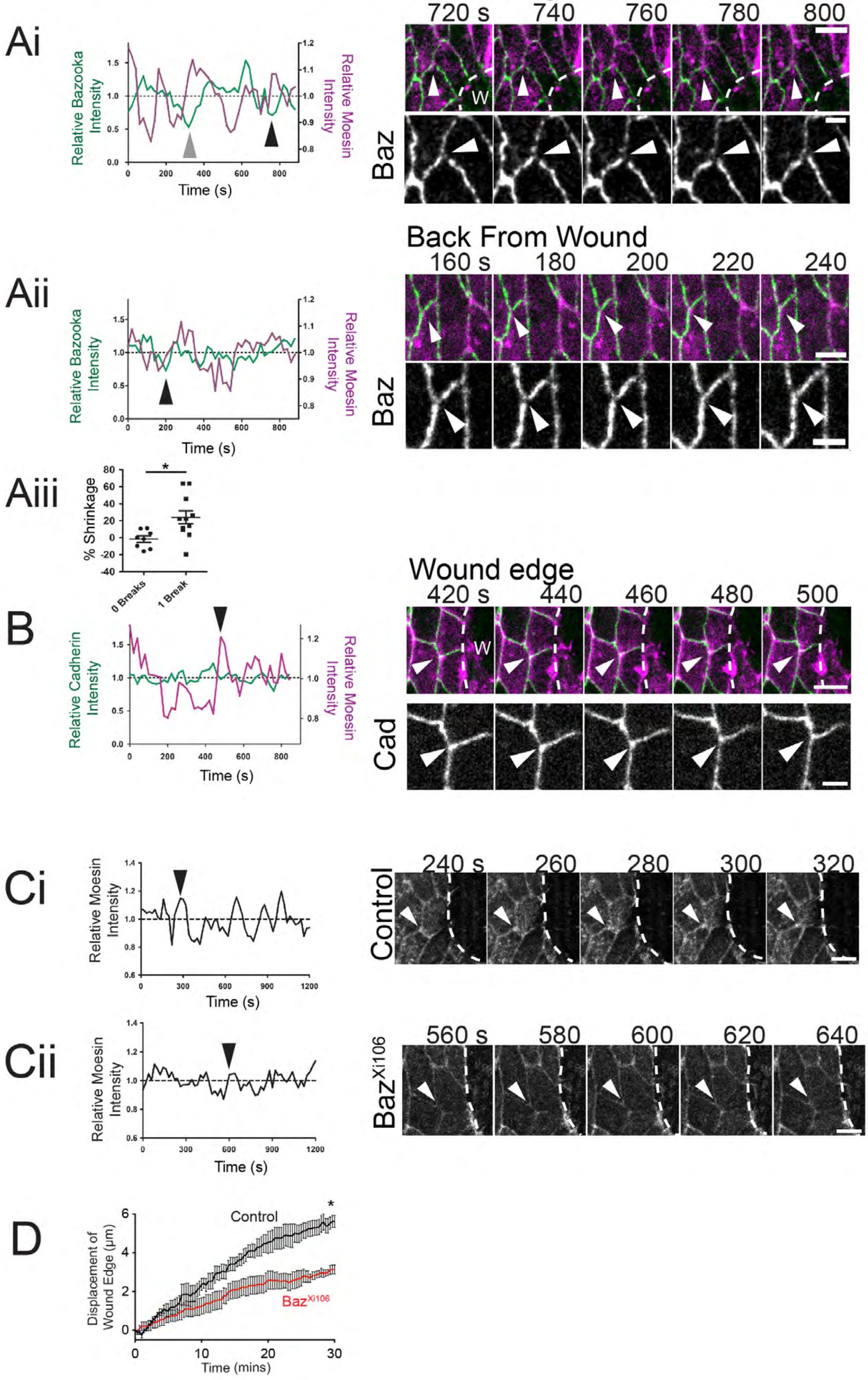
Ai
Relative Bazooka Intensity
Relative Moesin Intensity
Time (s)
Wound Edge
720 s
740
760
780
800
W
Baz
Aii
Back From Wound
160 s
180
200
220
240
Aiii
% Shrinkage
0 Breaks
1 Break
B
Relative Cadherin Intensity
Wound edge
420 s
440
460
480
500
Cad
Ci
Control
240 s
260
280
300
320
Cii
Baz^{Xi106}
560 s
580
600
620
640
D
Displacement of Wound Edge (µm)
Time (mins)
Control

Supplementary Figure S3

(Ai) Graph shows relative intensity of Bazooka-GFP (green) and mCherry-moesin (magenta) over several minutes of imaging showing multiple breaks at the same vertex. The grey arrowhead on the graph points to the break shown in Fig. 3Ei and the black arrowhead points to the break represented in Fig. S3Ai. The second break in Bazooka-GFP (green, and grey in the bottom panel) is shown in the accompanying still images and indicated by the arrowheads (mCherry-moesin shown as magenta). (Aii) A related graph to Ai but showing no breaks in vertices back from the wound edge. The small decrease in Bazooka-GFP (green, and grey in bottom panels) intensity at the vertex (highlighted by the arrowhead) is shown in the accompanying time series stills and shows no break in Bazooka. (Aiii) Graph of junction shrinkage of AP junctions associated with vertices showing either no Bazooka break or one break (vertices from 6 independent wounds). (B) Graph shows a representative plot of E-cadherin-GFP intensity at a vertex of a junction that recruits a pulse of actin (arrowhead) but is not preceded by a break in E-cadherin. The time points surrounding the actin pulse are shown in the accompanying time-lapse stills (E-cadherin-GFP in green, and grey in bottom panels, mCherry-moesin in magenta). (Ci-Cii) Graphs represents relative Moesin-GFP intensity at a vertex in a control embryo versus a Baz^{Xi106} mutant embryo. Peaks of Moesin-GFP intensity represent pulses of actomyosin at the vertex, the number of which are reduced in Baz^{Xi106} mutants. Arrowheads on graphs indicate the time points shown in the accompanying time-lapse stills showing an actin pulse at vertices (arrowheads) in control embryos but not in Baz^{Xi106} mutants. (D) Graph representing wound edge advancement in Control (black) vs Baz^{Xi106} mutant (red) embryos ($n \geq 5$ wounds for each genotype). Wound (W) margins marked by dashed lines. Times are in seconds for all. Scale bars represent 5 μm in all except in close up views of Bazooka-GFP and E-cadherin-GFP at the vertices which represent 2 μm. Error bars represent SEM. * denotes $p<0.05$ from a Student's t-test in Aiii and via a Two-way ANOVA with a Bonferroni post test in D.

Figure S4

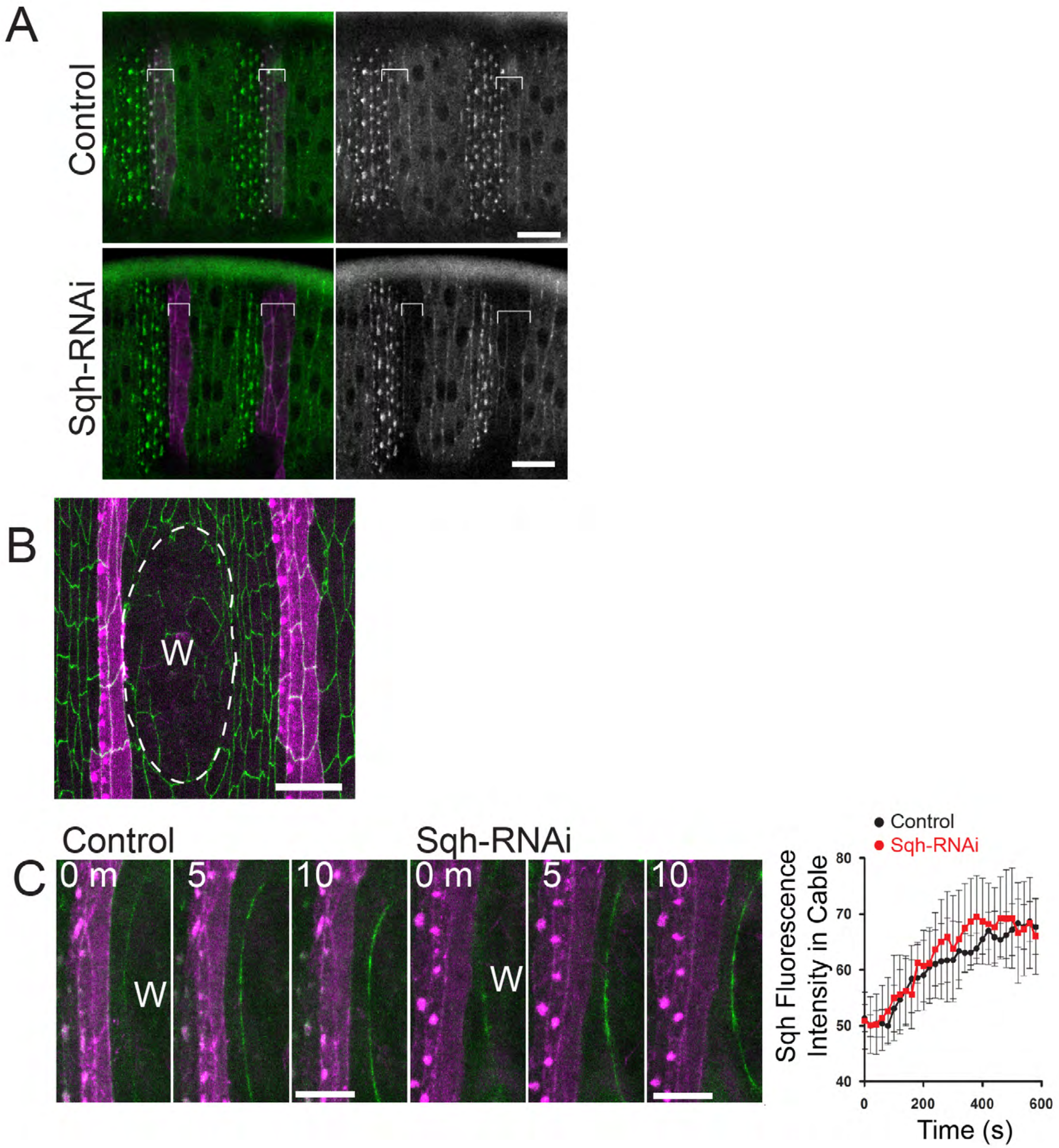

Supplementary Figure S4

(A) Images from a live embryo either control or expressing Sqh-RNAi in engrailed stripes (magenta) and Sqh-GFP (green) to show knockdown of Sqh in engrailed cells but not in cells adjacent. (B) Timelapse stills of Sqh-GFP (green) as it assembles a contractile cable in front row cells to show no difference in cable formation and that Sqh dynamics are not altered in neighbouring cells when Sqh-RNAi is expressed in engrailed stripes (magenta). The associated graph shows quantification of Sqh intensity of the cable revealing no difference between control embryos and Sqh-RNAi engrailed expressing embryos (n = 5 wounds for each). (C) A Low magnification example of the wounds made in E-cadherin-GFP (green) engrailed-mCherry-moesin (magenta) embryos to show that the wound edge does not encroach on more anterior engrailed domains. Wound positions indicated by “W”. Times are in minutes. Error bars represent SEM. Scale bars represent 15 μm in A, 10 μm in B and 20 μm in C.

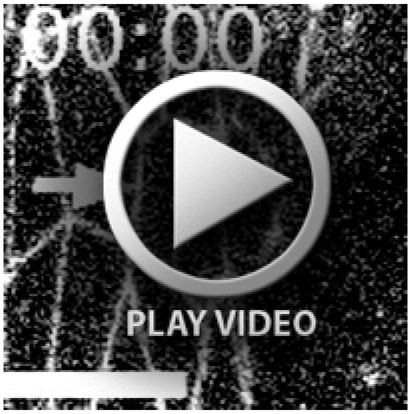

Supplementary Movie 1, related to Figure 2D. Junction shrinking occurs in a pulsatile manner. Timelapse movie of an E-cadherin-GFP wounded embryo showing that the shrinking junction (highlighted by the arrow) between cell rows one and two undergoes multiple rounds of contraction. Scale bar = 10 μm. Time is in minutes.

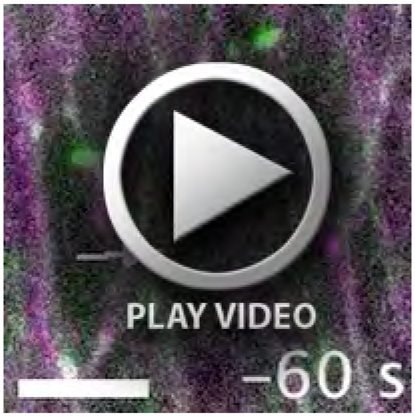

Supplementary Movie 2, related to Figure 3A. An actomyosin pulse in cell away from the wound edge. An mCherry-moesin (magenta), Sqh-GFP (green) expressing embryo showing that the stochastic pulses of myosin (arrow) in cells away from the wound edge occur at the apex of the cell. Scale bar = 5 μm. Time is in seconds.

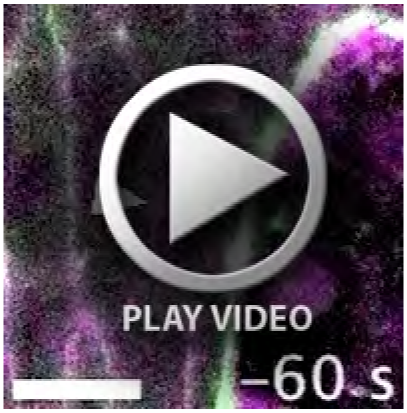

Supplementary Movie 3, related to Figure 3B. An actomyosin pulse in a cell at the wound edge. An mCherry-moesin (magenta), Sqh-GFP (green) wounded embryo showing the position of the myosin pulses (arrow) in wound edge cells is at the vertex of the shrinking AP junction (marked by the white arrowhead at the start of the movie). Scale bar = 5 μm. Time is in seconds.

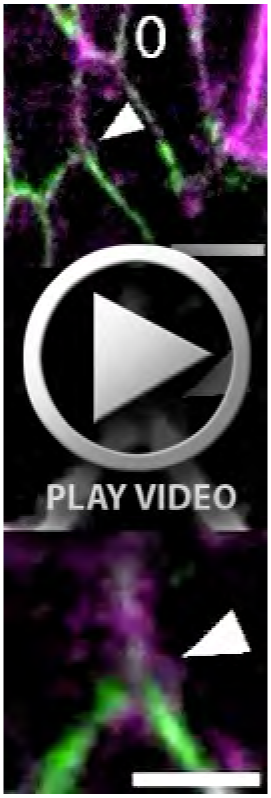

Supplementary Movie 4, related to Figure 3E. Bazooka break and actin pulse in a cell at the wound edge. A Bazooka-GFP (green, and grey in high magnification panels) and mCherry-moesin (magenta) wounded embryo showing a break in the Bazooka domain (arrowheads) recruits a pulse of actin that contracts the area surrounding the break. Scale bar = 5 µm in the top panel and 2 µm in the bottom two panels. Time is in seconds.

Table S1. Genotypes used in this study

Figure	Genotype
1A	w;; Da-Gal4, UAS-GMA/+
1B-D	w; Ubi-E-cadherin-GFP/ Crq-gal4, UAS-red stinger
2A-Div	w; Ubi-E-cadherin-GFP/ Crq-gal4, UAS-red stinger
3A-Diii	Sqh^{AX3}/Y; e22c-Gal4, UAS-mCherry-moesin/+; Sqh::Sqh-GFP/+
3Ei-iv	UAS-Bazooka-GFP/Y; e22c-Gal4, UAS-mCherry-moesin/+
3Fi Control	Baz^{Xi106}/FM7a dfd-YFP; Ubi-E-cadherin-GFP /+
3Fi Baz^{Xi106}	Baz^{Xi106}/Y; Ubi-E-cadherin-GFP /+
3Fii Control	Baz^{Xi106}/FM7a dfd-YFP;; Da-Gal4, UAS-GMA/+
3Fii Baz^{Xi106}	Baz^{Xi106}/Y;; Da-Gal4, UAS-GMA/+
4Ai-ii Control moe	Sqh^{AX3}/Y; e22c-Gal4, UAS-mCherry-moesin/+; Sqh::Sqh-GFP/+
4Aii Control cad	w; En-Gal4, UAS-mCherry-moesin, Ubi-E-cadherin-GFP/CyO; MKRS/ TM6b x y w;;
4Aii Sqh RNAi cad	w; En-Gal4, UAS-mCherry-moesin, Ubi-E-cadherin-GFP/CyO; MKRS/ TM6b x y sc v; P(TRiP.HMS00830)attP2
4B-C Control	w; En-Gal4, UAS-mCherry-moesin, Ubi-E-cadherin-GFP/CyO; MKRS/ TM6b x y w;;
4B-C Sqh RNAi	w; En-Gal4, UAS-mCherry-moesin, Ubi-E-cadherin-GFP/CyO; MKRS/ TM6b x y sc v; P(TRiP.HMS00830)attP2
S1Ai-Dii	w; Ubi-E-cadherin-GFP/ Crq-gal4, UAS-red stinger
S2Ai-Dii	Sqh^{AX3}/Y; e22c-Gal4, UAS-mCherry-moesin/+; Sqh::Sqh-GFP/+
S3Ai-iii	UAS-Bazooka-GFP/Y; e22c-Gal4, UAS-mCherry-moesin/+
S3B	w; Ubi-E-cadherin-GFP/ e22c-Gal4, UAS-mCherry-moesin
S3Ci Control	Baz^{Xi106}/FM7a dfd-YFP;; Da-Gal4, UAS-GMA/+
S3Ci Baz^{Xi106}	Baz^{Xi106}/Y;; Da-Gal4, UAS-GMA/+
S3D Control	Baz^{Xi106}/FM7a dfd-YFP; Ubi-E-cadherin-GFP /+
S3D Baz^{Xi106}	Baz^{Xi106}/Y; Ubi-E-cadherin-GFP /+
S4A and C Control	w; En-Gal4, UAS-mCherry-moesin /+; Sqh::Sqh-GFP/MKRS x y w;;
S4A and C Sqh-RNAi	w; En-Gal4, UAS-mCherry-moesin /+; Sqh::Sqh-GFP/MKRS x y sc v; P(TRiP.HMS00830)attP2
S4B	w; En-Gal4, UAS-mCherry-moesin, Ubi-E-cadherin-GFP/CyO; MKRS/ TM6b x y w;;
